# Long noncoding RNA DLGAP1-AS1 promotes the progression of glioma by regulating the miR-1297/EZH2 axis

**DOI:** 10.18632/aging.202923

**Published:** 2021-04-26

**Authors:** Liang Liu, Xiaojian Li, Yan Shi, Hua Chen

**Affiliations:** 1 Department of Neurosurgery, Nanjing First Hospital, Nanjing Medical University, Nanjing 210006, China; 2 Department of Neurosurgery, The First Affiliated Hospital of Nanjing Medical University, Nanjing 210029, China

**Keywords:** DLGAP1-AS1, miR-1297, EZH2, glioma

## Abstract

Dysregulated lncRNAs have been implicated in a plethora of tumors, including glioma. One such oncogenic lncRNAs that has been reported in several cancers is the lncRNA DLGAP1 antisense RNA 1 (DLGAP1-AS1). This study seeks to characterize the expression of DLGAP1-AS1 in glioma tissues, which we found to be raised in both glioma samples and cell lines. Functional experiments revealed that DLGAP1-AS1 promoted *in vitro* glioma cell invasion, migration and proliferation. DLGAP1-AS1 was found to function as a miR-1297 sponge, based on information from luciferase reporter assays, RNA pull-down assays and publicly available online databases. miR-1297 was in turn found to functionally target EZH2. DLGAP1-AS1 modulated EZH2 expressions through miR-1297 sponging. Glioma progression appears to be supported DLGAP1-AS1 -promoted activation of the miR-1297/EZH2 axis. The components of this axis may function as therapeutic targets for glioma.

## INTRODUCTION

The most frequently encountered primary central nervous system tumor diagnosed in adults often originate from the glial cells [1, 2]. Glioblastoma multiforme (GBM, glioma WHO grade IV) is the most malignant type of glioma [3]. At present, aggressive surgery combined with radiotherapy and chemotherapy still cannot satisfactorily prolong median patient survival [4]. The morbidity rates of GBM are high, and the five-year survival rate remains below 3%, largely owing to the resistance to chemotherapy and radiotherapy, as well as its malignant invasion and rapid proliferation [5]. A deeper grasp of the underlying biological pathways in glioma represents a key step in the discovery of more potent therapeutic mechanisms.

Long noncoding RNAs (lncRNAs) are a non-protein coding section of the genome measuring approximately 200 nucleotides in length [6, 7]. These molecules can regulate expression of genes at the apparent, transcriptional, and post-transcriptional levels through a variety of interactions between DNA, RNA and proteins [8, 9]. These molecules have received much attention with regards to their role in the initiation and development of several diseases. Wu et al. reported that temozolomide (TMZ) resistance in glioma appears to be induced by lnc-TALC regulation on the c-Met pathway [10]. Zheng et al. highlighted the enhancing effect of lncRNA NNT-AS1 on glioma cell proliferation and metastasis through its action on the miR-494-3p/PRMT1 axis [11]. In addition, Dong et al. demonstrated that lncRNA EGFR-AS1 is involved in glioma cell apoptosis, invasion and migration [12]. DLGAP1 antisense RNA 1 (DLGAP1-AS1) is located on chromosome 18p11.31, and has previously been reported to be closely related to various tumours. Deng et al. reported that gastric cancer progression is influenced by the DLGAP1 molecule via its effects on the miR-628-5p/AEG-1 axis [13]. In hepatocellular cancer, Lin et al. implicated DLGAP1-AS1 expression in epithelial-mesenchymal transformation and tumor development [14]. Despite the wealth of information available, little has been reported regarding its role in the context of glioma.

MicroRNAs (miRNAs) represent 22 nucleotide long non-coding single-stranded RNA molecules which are able to modulate genetic expression in a post-transcriptional manner [15]. The role of miRNAs in the diagnosis and prognosis of glioma has been recognized by many studies [16]. As a member of miRNA family, miR-1297 has been studied in various cancers, including glioma [17, 18], however, the related molecular mechanism has not been fully understood. The Enhancer of zeste homologue 2 (EZH2) is a catalytic subunit of the polycomb repressive complex 2 which has been found to be actively involved across several different cancers [19, 20]. In glioma, studies have shown that EZH2 could be regulated by many miRNAs, such as miR-9 [21], miR-133b [22] and miR-1265 [23]. However, its regulatory relationship with miR-1297 has not been studied.

In this study, we sought to characterize the expression, biological function and regulatory mechanism of DLGAP1-AS1, miR-1297 and EZH2 in glioma. These findings may serve to provide new insights into the biological workings of this debilitating disease.

## RESULTS

### An upregulation of DLGAP1-AS1 in glioma tissues and cell lines

**Figure 1:**
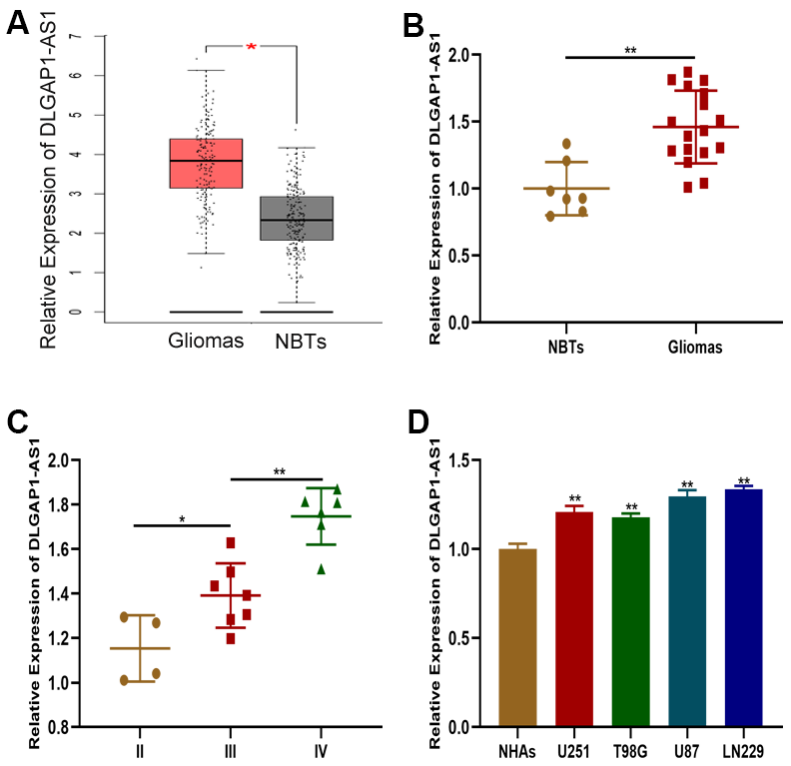
**DLGAP1-AS1 expression was upregulated in glioma tissues and cell lines.** (**A**) Expression of DLGAP1-AS1 in a public database. (**B**) Expression of DLGAP1-AS1 in normal brain tissues (n=7) and glioma tissues (n=17). (**C**) Expression of DLGAP1-AS1 in different grades of glioma. (**D**) Expression of DLGAP1-AS1 in normal human astrocytes and glioma cell lines. *p < 0.05, **p < 0.01.

The GEPIA database was first searched to obtain further insights into DLGAP-AS1 expression in GBM (Figure 1A). DLGAP1-AS1 expression was then characterized using qRT-PCR in glioma tissues (n=17) and normal brain tissues (n=7). DLGAP1-AS1 was found to be more highly expressed in glioma tissues in contrast to healthy brain tissues (Figure 1B). These findings were similar to those extracted from the GEPIA database. Glioma grade was found to be associated to DLGAP1-AS1 was expression (Figure 1C). qRT-PCR analysis of DLGAP1-AS1 expression in three different glioma cell lines (U251, T98G, U87, and LN229) and NHAs found that the expression of this molecule was markedly raised in glioma cell lines in contrast to NHAs. This finding was particularly prominent in the LN229 and U87 cell lines (Figure 1D). Glioma development may be critically modulated by DLGAP1-AS1 expression.

### Silencing DLGAP1-AS1 suppressed glioma cell invasion, migration and proliferation

**Figure 2:**
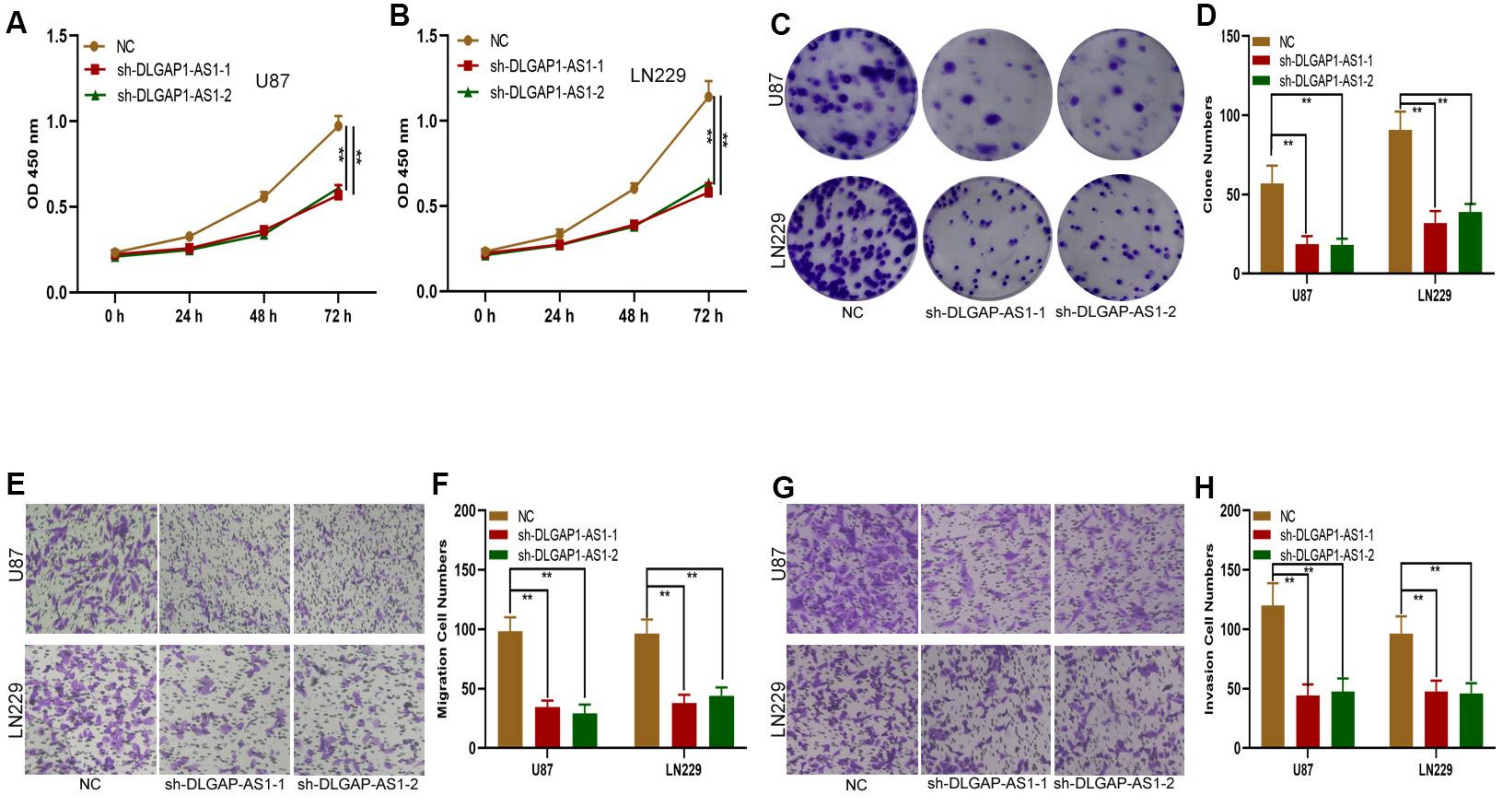
**Silencing DLGAP1-AS1 inhibited proliferation, migration and invasion in glioma cells.** (**A**, **B**) CCK-8 assays showed the proliferative capacity of the glioma cell lines transfected with NC, sh-DLGAP1-AS1-1 or sh-DLGAP1-AS1-2. (**C**, **D**) Clone formation assays showed the proliferative capacity of the glioma cell lines transfected with NC, sh-DLGAP1-AS1-1 or sh-DLGAP1-AS1-2. (**E**, **F**) Transwell assays showed the migratory capacity of the glioma cell lines transfected with NC, sh-DLGAP1-AS1-1 or sh-DLGAP1-AS1-2. (**G**, **H**) Transwell assays showing the invasive capacity of the glioma cell lines transfected with NC, sh-DLGAP1-AS1-1 or sh-DLGAP1-AS1-2. *p < 0.05, **p < 0.01.

In order to determine the impact of DLGAP1-AS1 on glioma progression, we transfected shRNAs targeting DLGAP1-AS1 (sh-DLGAP1-AS1-1 and sh-DLGAP1-AS1-2) into LN229 and U87 cells. Transfection efficiency was validated using qRT-PCR (Supplementary Figure 1A, 1B). Subsequent CCK-8 assays demonstrated that knockdown of DLGAP1-AS1 notably inhibited the proliferative abilities of U87 and LN229 cells (Figure 2A, 2B). Consistent with the CCK-8 assay, knockdown of DLGAP1-AS1 resulted in smaller colony formation by both U87 and LN229 cells (Figure 2C, 2D). DLGAP1-AS1 silencing suppressed glioma cell invasion and migration, as demonstrated by transwell assays (Figure 2E, 2H). Our findings support the roles of DLGAP1-AS1 in regulating the invasive, migratory and proliferative abilities of glioma cells.

### Silencing DLGAP1-AS1 inhibited tumorigenicity *in vivo*

**Figure 3:**
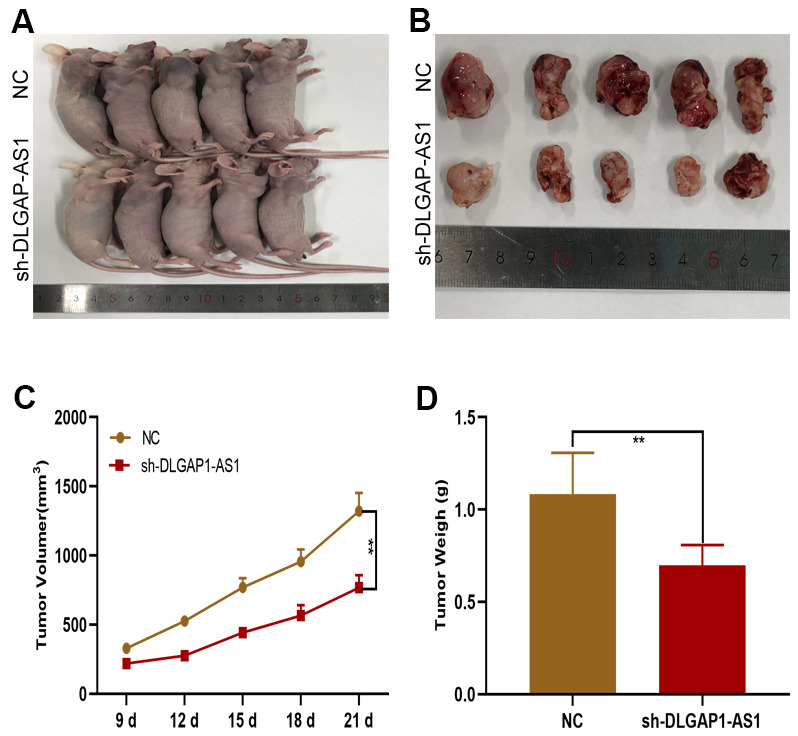
**DLGAP1-AS1 promoted glioma growth *in vivo*.** (**A**, **B**) Tumour formation in nude mice. (**C**, **D**) Silencing of DLGAP1-AS1 led to a reduction in tumour growth (including volume and weight) in nude mice compared to that in the control group. *p < 0.05, **p < 0.01.

A subcutaneous xenograft model was generated for investigation of the *in vivo* effects of DLGAP1-AS1 on tumour development. sh-DLGAP1-AS1 (including sh-DLGAP1-AS1-1 and sh-DLGAP1-AS1-2) inhibited xenograft tumour growth in contrast to control mice (Figure 3A, 3B). Mean tumor weights and volumes were also lower in the sh-DLGAP1-AS1 group (Figure 3C, 3D). These data suggested that silencing DLGAP1-AS1 inhibited *in vivo* tumour growth.

### DLGAP1-AS1 functions as a miR-1297 sponge

**Figure 4:**
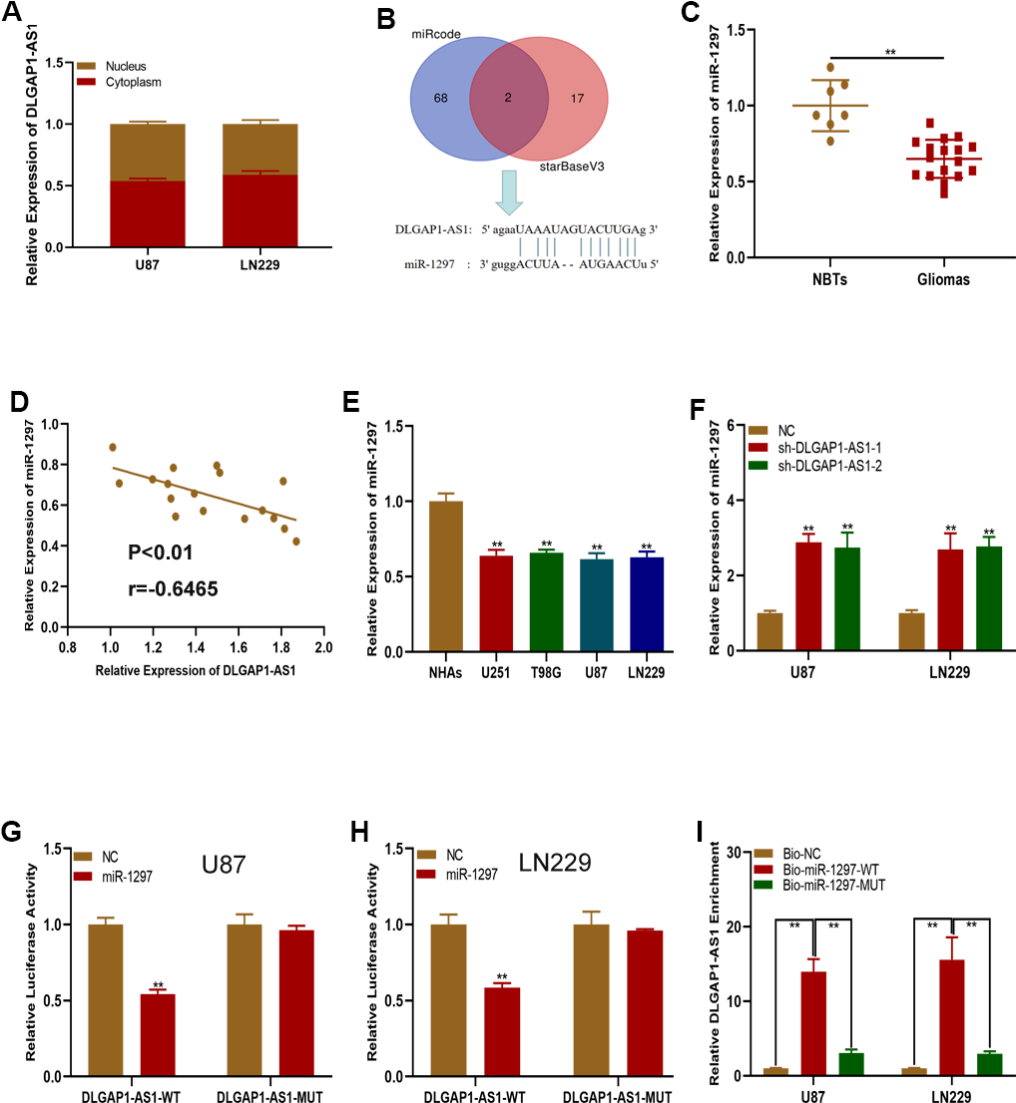
**DLGAP1-AS1 functions as a sponge of miR-1297.** (**A**) qRT-PCR assays showed the expression of DLGAP1-AS1 in the cytoplasm and nucleus. (**B**) MiR-1297 was predicted as a target of DLGAP1-AS1 through online bioinformatic databases. (**C**) Expression of miR-1297 in normal brain tissues (n=7) and glioma tissues (n=17) based on qRT-PCR. (**D**) Pearson's correlation analysis indicated that there is negative regulation between DLGAP1-AS1 and miR-1297 in glioma tissues. (**E**) Expression of miR-1297 in normal human astrocytes and glioma cell lines based on qRT-PCR. (**F**) qRT-PCR analysis showed that miR-1297 was negatively regulated by DLGAP1-AS1. (**G**, **H**) Luciferase reporter assays showed that miR-1297 reduced the luciferase activity of DLGAP1-AS1-WT but not of DLGAP1-AS1-MUT. (**I**) RNA pull-down assays demonstrated that biotagged wild-type DLGAP1-AS1 was enriched for miR-1297, while the empty vector and biotagged mutant-type DLGAP1-AS1 were not. *p < 0.05, **p < 0.01.

We then sought to characterize the underlying molecular mechanisms of DLGAP1-AS1 in glioma. A subcellular fractionation location assay found DLGAP1-AS1 to be primarily expressed in glioma cell cytoplasm (Figure 4A), highlighting the potential role of this molecule as a ceRNAs. Online bioinformatics databases (miRcode and starBase) were utilize to determine potential miRNAs of DLGAP1-AS1 target gene, with the miR-1297 being of special interest (Figure 4B). qRT-PCR showed that miR-1297 was more highly expressed in healthy brain samples in contrast to glioma tissues (Figure 4C). There appeared to be a negative correlation between DLGAP1-AS1 and miR-1297 in glioma tissues, as determined using Pearson correlation analysis (Figure 4D). miR-1297 quantification in glioma and NHAs found that it was of lower expression in glioma cell lines (Figure 4E). Interestingly, raised miR-1297 expression was characterized in U87 and LN229 cells transfected with sh-DLGAP1-AS1 (Figure 4F). Moreover, miR-1297 mimic transfection into the U87 and LN229 cells suppressed DLGAP1-AS1-WT luciferase activities, but not those of DLGAP1-AS1-MUT (Figure 4G, 4H). An RNA pull-down assays revealed that biotagged wild-type DLGAP1-AS1 possessed high expressions of miR-1297, findings which were not seen in biotagged mutant-type DLGAP1-AS1 and empty vectors (Figure 4I). The above findings suggested that DLGAP1-AS1 could sponge miR-1297.

### MiR-1297 targeted EZH2 to suppress glioma cell proliferation, migration and invasion

**Figure 5:**
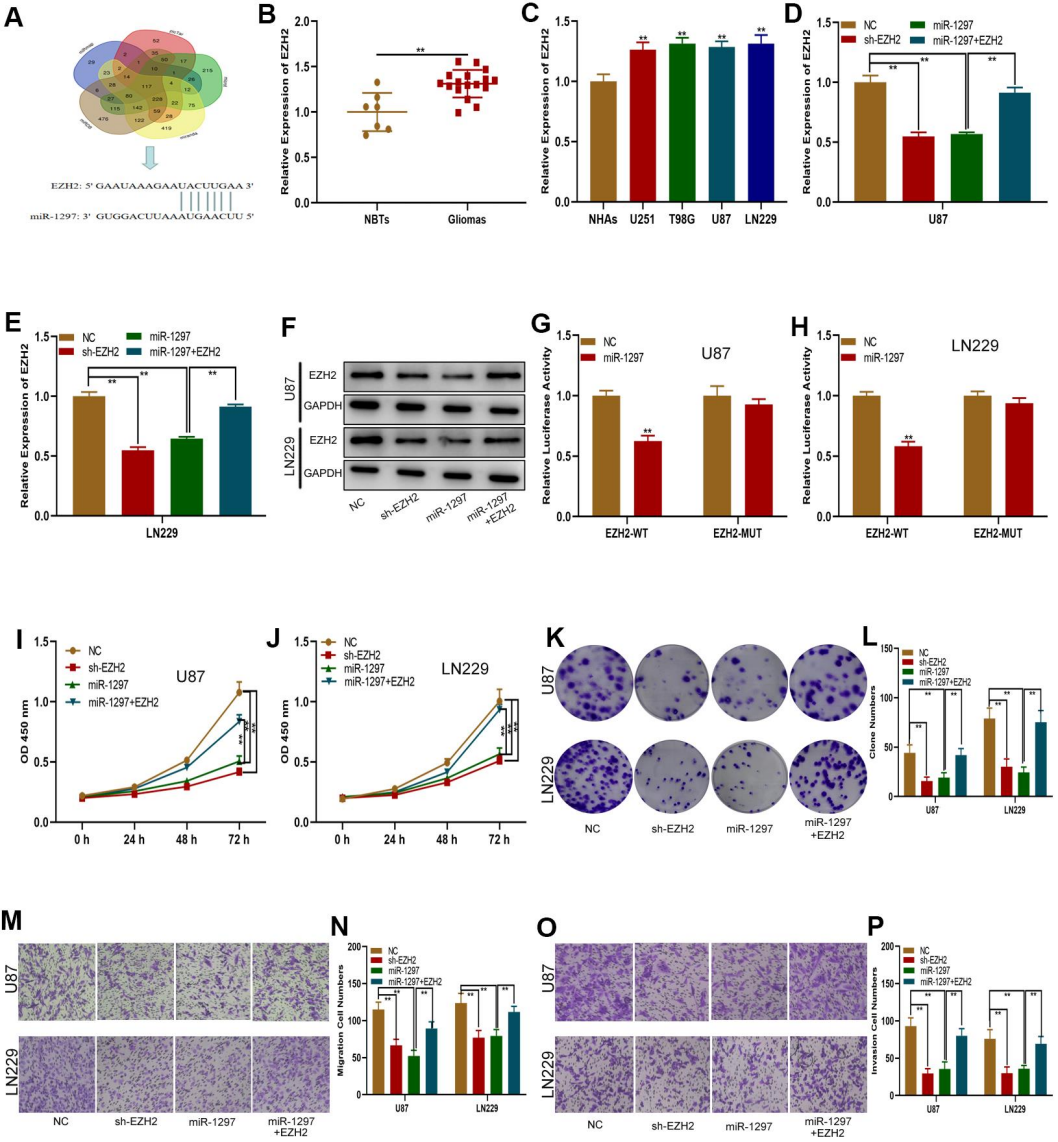
**MiR-1297 inhibited proliferation, migration and invasion in glioma cells by targeting EZH2.** (**A**) EZH2 was predicted as a target of miR-1297 through online bioinformatic databases. (**B**) Expression of EZH2 in normal tissues (n=7) and glioma tissues (n=17). (**C**) Expression of EZH2 in normal human astrocytes and glioma cell lines based on qRT-PCR. (**D**–**F**) qRT-PCR and western blotting showing the expression of EZH2 in glioma cell lines transfected with NC, sh-EZH2, miR-1297 or miR-1297 together with the EZH2 plasmid. (**G**, **H**) Luciferase reporter assays showed that miR-1297 reduced the luciferase activity of EZH2-WT but not of EZH2-MUT. (**I**, **J**) CCK-8 assays showing the proliferative capacity of glioma cell lines transfected with NC, sh-EZH2, miR-1297 or miR-1297 together with the EZH2 plasmid. (**K**, **L**) Colony formation assays showing the proliferative capacity of glioma cell lines transfected with NC, sh-EZH2, miR-1297 or miR-1297 together with the EZH2 plasmid. (**M**–**P**) Transwell assays showing the migration and invasion of glioma cell lines transfected with NC, sh-EZH2, miR-1297 or miR-1297 together with the EZH2 plasmid. *p < 0.05, **p < 0.01.

To verify this ceRNA hypothesis, we first sought to determine the miR-1297 target gene. Online database searches revealed EZH2 to be a downstream target gene of miR-1297 (Figure 5A). EZH2 possessed raised expressions in glioma tissues and cells in comparison to NHAs and normal brain tissues, as shown by qRT-PCR analysis (Figure 5B, 5C). EZH2 expression was detected in glioma cells transfected with sh-EZH2, miR-1297 mimics or miR-1297 mimics in combination with the EZH2 plasmid (Figure 5D–5F). Both sh-EZH2 and miR-1297 mimics suppressed EZH2 expression, with exposure to the EZH2 plasmid partially reversing the inhibitory impact of miR-1297 mimics. In addition, luciferase reporter assays revealed that luciferase activity of EZH2-WT was inhibited by miR-1297, however, no effects was observed in EZH2-MUT (Figure 5G, 5H). Our findings highlight that EZH2 may function as the target gene of miR-1297. Additional, transwell, colony formation, and CCK-8 assays were performed and revealed that the impact of miR-1297 mimics on glioma cell invasion, migration and proliferation was reversed upon exposure to the EZH2 plasmid (Figure 5I–5P). In summary, miR-1297 appeared to inhibit the invasive, migratory and proliferative capabilities of glioma cells through its action on EZH2.

### DLGAP1-AS1 indirectly regulated the expression of EZH2 and enhanced the proliferation, migration and invasion of glioma cells through miR-1297 sponging

**Figure 6:**
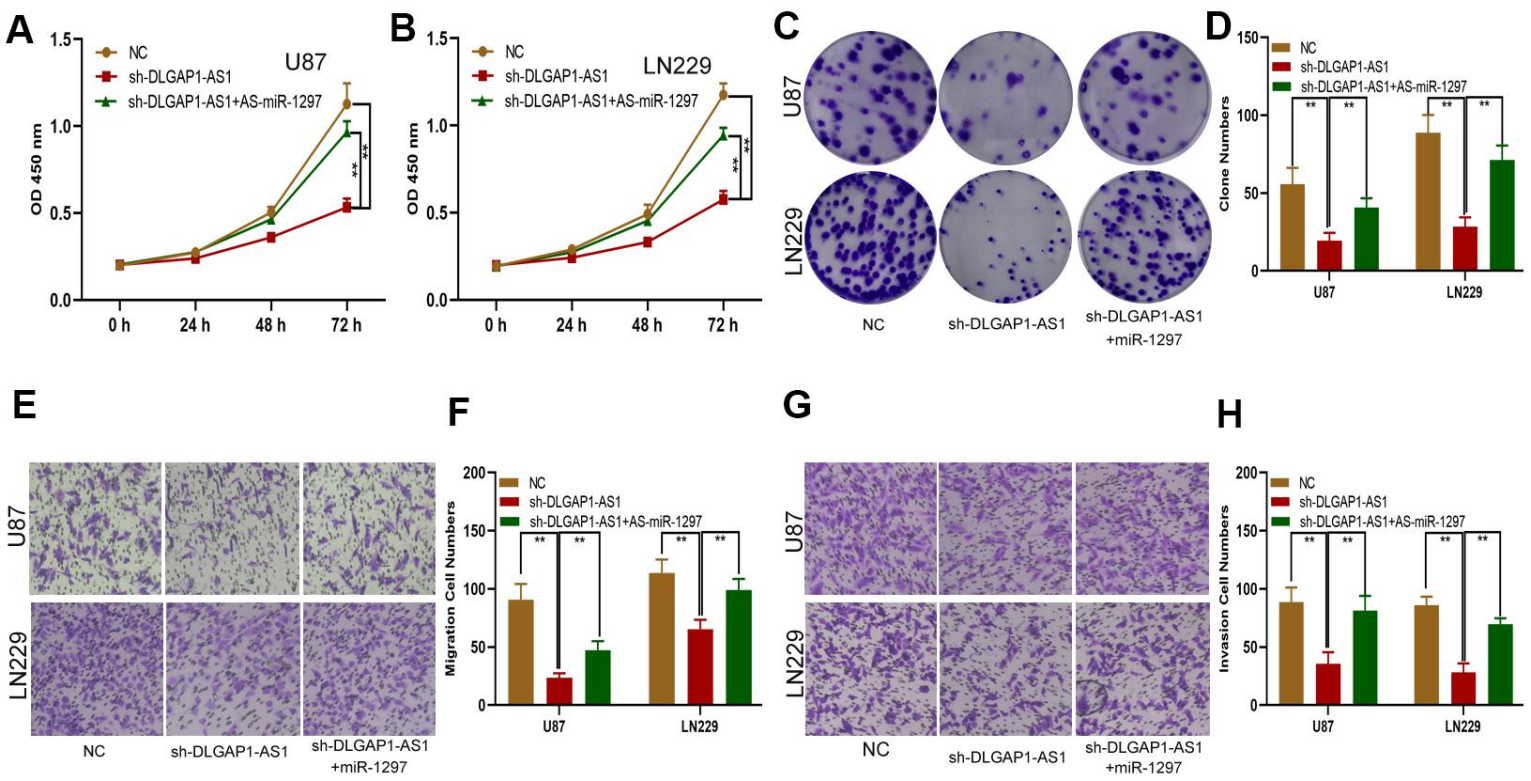
**DLGAP1-AS1 promoted proliferation, migration and invasion in glioma cells by sponging miR-1297 and indirectly regulating EZH2 expression.** (**A**, **B**) CCK-8 assays showing the proliferative capacity of the glioma cell lines transfected with NC, sh-DLGAP1-AS1 or sh-DLGAP1-AS1 together with miR-1297 inhibitors. (**C**, **D**) Colony formation assays showing the proliferative capacity of the glioma cell lines transfected with NC, sh-DLGAP1-AS1 or sh-DLGAP1-AS1 together with miR-1297 inhibitors. (**E**–**H**) Transwell assays showing the migration and invasion of the glioma cell lines transfected with NC, sh-DLGAP1-AS1 or sh-DLGAP1-AS1 together with miR-1297 inhibitors. *p < 0.05, **p < 0.01.

We sought to explore the ceRNA function of DLGAP1-AS1 and whether it could sponge miR-1297 to regulate EZH2. First, EZH2 expression in sh-DLGAP1-AS1 transfected glioma cells (including sh-DLGAP1-AS1-1 and sh-DLGAP1-AS1-2) or sh-DLGAP1-AS1 together with miR-1297 inhibitors was explored using western blots and qRT-PCR. Downregulation of DLGAP1-AS1 appeared to suppress EZH2, with these effects partially reversed through the use of miR-1297 inhibitors (Supplementary Figure 1C, 1D). Subsequently, functional experiments demonstrated similar effects by the use of miR-1297 inhibitors in sh-DLGAP1-AS1 (Figure 6). DLGAP1-AS1 could enhance glioma cell proliferation, migration and invasion by sponging miR-1297 to regulate EZH2.

## DISCUSSION

Several studies have reported the close association of aberrant lncRNAs and miRNAs expressions to tumor development, including glioma [24, 25]. lncRNA HAS2-AS1 promoted tumour progression in glioma by acting as a ceRNA [26]. HCG11 modulated glioma progression through its action on the miR-496/CPEB3 axis [27]. Moreover, DLGAP1-AS1 has also been implicated in several processes involved in tumor progression [24, 25]. However, the expression and functional role of DLGAP1-AS1 in glioma has yet to be completely characterized. This investigation seeks to clarify the impact and hypothesized molecular function of DLGAP1-AS1 in glioma progression. DLGAP1-AS1 expression appears to be increased in glioma cell lines and tissues. In addition, our experimental data showed that DLGAP1-AS1 promoted the *in vitro* invasive, migratory and proliferative abilities of glioma cells.

Evidence points towards the likely role of lncRNAs as miRNA sponges in modulation of their target genes [28, 29]. DLGAP1-AS1 was localized in the cytoplasm of In U87 and LN229 cells, suggesting that it may function as a miRNA decoy or sponge to regulate target gene expression. Through analysis of data available on an online database, miR-1297 was determined as a candidate downstream target of DLGAP1-AS1. Further experiments suggest that DLGAP1-AS1 may function to sponge miR-1297. Several reports have implicated the suppressive role of miR-1297 across several cancers. Li et al. demonstrated that mRNA-1297 reduces glioblastoma cancer cell growth and metastasis through KPNA2 repression [30]. Zhang et al. documented that miR-1297 enhances gastric cancer cell apoptosis by downregulating CDC6 [31]. Our experimental data demonstrated that miR-1297 functions as a glioma tumour suppressor.

Enhancer of zeste homologue 2 (EZH2) is located on chromosome 7q36.1 and is involved in histone methylation and gene silencing through post-translational histone modifications [32, 33]. Our study highlights the oncogenic role of EZH2 in glioma progression which is in turn regulated by miR-1297. These findings are consistent with those previously reported [21, 34]. Furthermore, we show that DLGAP1-AS1 could regulate EZH2 expression and promote glioma cell invasion, migration and proliferation through miR-1297 sponging.

**Figure 7:**
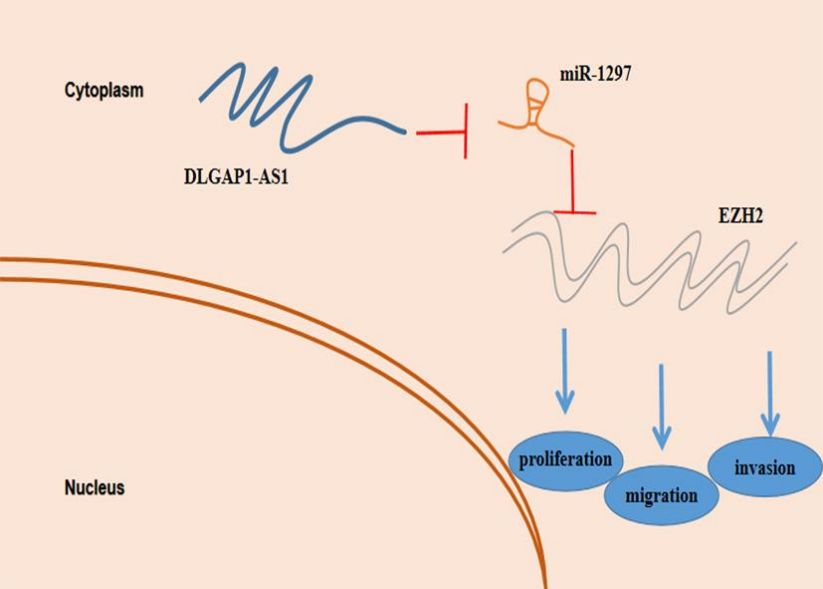
Schematic model depicting the function of ZFAS1 in proliferation, migration and invasion and reduced resistance to temozolomide in glioma.

Our study, for the first time, depicts DLGAP1-AS1 as a crucial oncogenic protein in glioma. DLGAP1-AS1 promoted glioma progression through miR-1297 sponging and EZH2 regulation (Figure 7). Our study highlights the existence of a novel biological axis involved in glioma which could serve as the basis for formulation of more advanced diagnostic and therapeutic methods.

## MATERIALS AND METHODS

### Human tissues

7 normal and 17 glioma tissue samples were procured from patients who underwent cranial decompression surgery and tumour resection at The First Affiliated Hospital of Nanjing Medical University, respectively. All brain specimens were immediately cryopreserved in liquid nitrogen. Ethical approval was gained by the Ethics Committee of The First Affiliated Hospital of Nanjing Medical University, with all patients providing written informed consent.

### Cell culture

The American Type Culture Collection (Manassas, VA, USA) provided glioma cell lines (U251, T98G, U87 and LN229) used for this experiment, while the JENNIO Biological Technology (Guangzhou, China) provided normal human astrocyte cell lines (NHAs). All cells were maintained in Dulbecco’s modified Eagle’s medium (DMEM, Gibco, NY, USA) supplemented with 10% foetal bovine serum (FBS, ScienCell, LA, USA), 100 U/mL penicillin and 100 μg/mL streptomycin. Incubation conditions were the following: 5% CO_2_ atmosphere and 37° C temperature.

### Plasmid constructs, oligonucleotides and cell transfection

Short hairpin RNAs (shRNAs) which targeted DLGAP1-AS1 (sh-DLGAP1-AS1-1 and sh-DLGAP1-AS1-2), shRNA targeting EZH2 (sh-EZH2) and their negative controls as well as Hsa-miR-1297 mimics, inhibitors and their negative controls were synthesized by GenePharma (Shanghai, China). The Lipofectamine 3000 (Invitrogen, Carlsbad, CA, USA) reagent was used to transfect the oligonucleotides into the U87 and LN229 cells using in compliance to instructions stipulated by the manufacturers.

### Extraction of RNA and quantitative RT-PCR (qRT-PCR)

The TRIzol (Invitrogen, Carlsbad, CA, USA) reagent was used to isolate total RNA from tissues and cells. The Fermentas reverse transcription reagent was used to construct cDNA, which was then subjected to qRT-PCR analysis with the SYBR Green PCR Master Mix (Applied Biosystems, Thermo Fisher Scientific, MA, USA) in compliance to manufacturer protocols. Gene expressions were normalized against GAPDH and U6 expression. All data was quantified using the 2^−ΔΔCt^ method. Table 1 depicts primer sequences used in these experiments.

**Table 1:** The primers used in this study.

**Gene**	**Primer sequence**
DLGAP1-AS1	F: 5′-GGGGCAGGAGTAAAGTGGAC-3′
	R: 5′-CCAGACATATAGCAGCCGGG-3′
miR-1297	F: 5'- ACACTCCAGCTGGGTCCTTCATTCCA -3′
	R: 5'- GTGCAGGGTCCGAGGT -3′
EZH2	F: 5’- CAAGAGGTTCAGACGAGCTGATG -3’
	R: 5’- CACAGGCTGTATCCTTCGCTG -3’
GAPDH	F: 5’- GTC AAC GGA TTT GGT CTG TATT-3’
	R: 5’- AGT CTT CTG GGT GGC AGT GAT-3′
U6	F: 5′-CTCGCTTCGGCAGCACA-3′
	R: 5′-AACGCTTCACGAATTTGCGT-3′
18S	F: 5′-GTAACCCGTTGAACC CCATT-3′
	R: 5′- CCAT CCAATCGGTAG TAGCG-3′

### Western blot analysis

Western blot analysis was carried out previously described [35]. Primary antibody against EZH2 at a dilution ratio of 1:1000 was purchased from Proteintech (IL, USA). HRP-conjugated secondary antibody at a dilution ratio of 1:2000 and GAPDH at a dilution ratio of 1:1000 were purchased from Proteintech (IL, USA).

### Subcellular fractionation

The subcellular fractionation of U87 and LN229 cells was performed with the PARIS™ Kit (Invitrogen, Carlsbad, CA, USA) to localize DLGAP1-AS1. The relative expression of DLGAP1-AS1 was measured in the extracted RNA by qRT-PCR. U6 and 18S were used as nuclear and cytoplasmic controls, respectively [36].

### Luciferase reporter assay

Bioinformatic websites were used to predict the role of miR-1297 in relation to DLGAP1-AS1 and EZH2. miR-1297 – interacting 3'-UTR fragments of DLGAP1-AS1 and EZH2 were constructed to interact with the pmir-report vector. Reporter constructs were cotransfected with the cells. A Dual Luciferase Reporter Assay System (Promega, WI, USA) was used to quantify luciferase according to the manufacturer’s instructions [37].

### RNA pull-down assay

Biotinylated miR-1297 was obtained from GenePharma (Shanghai, China) to produce biotinylated negative and mutant controls. U87 and LN229 cells were used to transfect biotinylated miR-1297 as well as biotinylated mutant and negative controls. M-280 streptavidin magnetic beads (Invitrogen, Carlsbad, CA, USA) was used to incubate the cell lysates. Extraction of the bound RNA was then performed followed by qRT-PCR detection of DLGAP1-AS1.

### CCK-8 assay

96-well plates (10,000 cells per well) were used to house cells which were then cultured for 24, 48, and 72 h. Cells were then exposed to 100 μL of Cell Counting Kit-8 (CCK-8, Beyotime, Shanghai, China) reagent for another 4 hour incubation period. A microplate reader was used to measure the absorbance value at 450 nm.

### Colony formation assay

All cells were cultured for 12 days in culture dishes (200 cells per dish). Phosphate buffered saline (PBS) was then used to rinse the cells twice before subjected to a 20 minute fixing period with 4% paraformaldehyde before being stained with 0.1% crystal violet for 15 min. Visible colonies were then counted.

### Migration assay and transwell assay

Cell migration and invasion were assessed using migration and Transwell assays. The invasion assay was performed using chamber inserts (Merck Millipore, Germany) coated with 50 μl of Matrigel (1:9 dilution; BD Bioscience, USA). The upper chambers for both assays were seeded with 10,000 cells in serum-free medium. The lower chambers contained DMEM with 10% FBS. Cells were then allowed to incubate 24 h at 37° C in a 5% CO_2_ incubator. Chambers were then fixed using 4% paraformaldehyde and stained for 30 mins with crystal violet solution before being rinsed thrice with PBS. An optical microscope was used to observe and count stained cells [38].

### Xenograft tumour assay

10 immunodeficient male nude mice (Beijing Laboratory Animal Centre, Beijing, China) of 5-6 weeks of age were reared under specific pathogen-free (SPF) environments. Two mice cohorts were formed (5 mice per group). Subcutaenous injections containing 1.0 × 10^7^ U87 cells stably expressing NC or sh-DLGAP1-AS1 were administered to the mice. Tumor volumes were measured every three days before the mice were sacrificed at day 21. Tumors were harvested and weighed.

### Statistical analysis

The SPSS 13.0 software was used for all data analysis. All data is depicted in terms of mean ± standard error. T tests or one-way ANOVA was used to analyse results. Statistical significance was conferred when P < 0.05 (*), and P < 0.01 (**) was considered to indicate high statistical significance. Results were the product of three independent experiments.

## Supplementary Material

Supplementary Figure 1
