# Supplementary material for: Long noncoding RNA DLGAP1-AS1 promotes the progression of glioma by regulating the miR-1297/EZH2 axis
Source: Aging (Albany NY). 2021 Apr 26;13(8):12129–42. doi: 10.18632/aging.202923 (PMC8109124; doi:10.18632/aging.202923)
Supplement: Supplementary Figure 1 [file aging-13-202923-s001.pdf]

SUPPLEMENTARY FIGURE

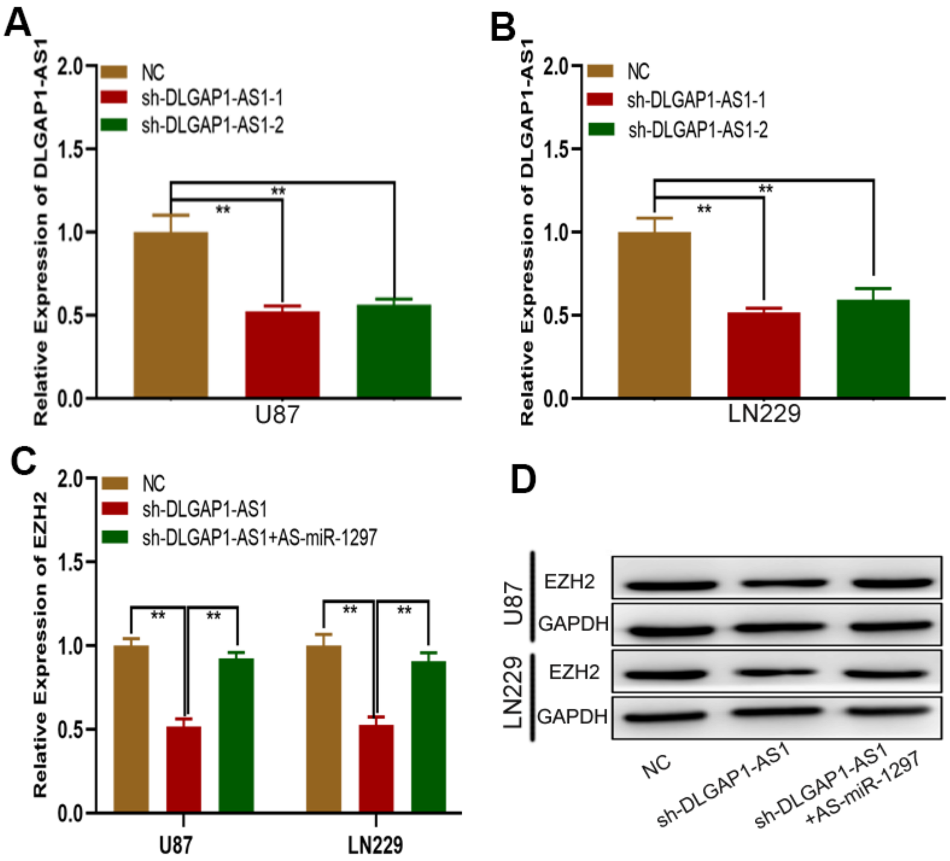

Supplementary Figure 1. (**A**, **B**) qRT-PCR analysis of DLGAP1-AS1 expression in U87 and LN229 cells transfected with NC, sh-DLGAP1-AS1-1 or sh-DLGAP1-AS1-2. (**C**, **D**) Expression of EZH2 in the cells transfected with NC, sh-DLGAP1-AS1 or sh-DLGAP1-AS1 together with miR-1297 inhibitors measured by qRT-PCR and western blotting.
